# Intensive Care Patients from the First COVID-19 Wave: One-Year Survival after Tocilizumab Treatment

**DOI:** 10.3390/jpm11111234

**Published:** 2021-11-21

**Authors:** Gabriele Melegari, Enrico Giuliani, Chiara Dallai, Lucia Veronesi, Elisabetta Bertellini, Suela Osmenaj, Alberto Barbieri

**Affiliations:** 1 Department of Anaesthesia and Intensive Care, Azienda Ospedaliero-Universitaria di Modena, 41121 Modena, Italy; chiaradallai88@gmail.com (C.D.); bertellini.elisabetta@aou.mo.it (E.B.); 2 Department of Medical, Surgical, Maternal-Child and Adult Sciences, University of Modena and Reggio Emilia, 41121 Modena, Italy; en.giuliani@gmail.com; 3 School of General Surgery, University of Modena and Reggio Emilia, 41121 Modena, Italy; veronesi.lucia@gmail.com; 4 School of Anaesthesia and Intensive Care, University of Modena and Reggio Emilia, 41121 Modena, Italy; osmenajsuela@gmail.com (S.O.); alberto.barbieri@unimore.it (A.B.)

**Keywords:** COVID-19, cytokine storm medication, COVID-19 post-infection symptoms

## Abstract

Introduction: An infection by COVID-19 triggers a dangerous cytokine storm, so tocilizumab has been introduced in Italy as an agent blocking the cytokine storm. This paper aims to describe the one-year survival of ICU patients treated with tocilizumab. Methods: This observational study enrolled all patients confirmed to be infected by COVID-19 who were admitted to the ICU in our center. We offered tocilizumab to all non-septic patients if they did not present any contraindications. Results: We enrolled 68 ICU patients in our center on 72 occasions during the enrollment period; we excluded four patients due to study criteria. The one-year mortality hazard ratio of treated patients was 0.64, with a confidence interval of 0.31 to 1.19, with *p* = 0.169. Among the survivors, 32 of 35 patients answered the phone interview (14 patients in the treated group and 18 in the untreated group); overall, the effect of COVID-19 on quality of life was 58.14%. These effects were lower in the tocilizumab group, with *p* = 0.016 *. Conclusions: Our observational data follow the most relevant largest trial. Patients treated with tocilizumab had lower rates of new-onset symptoms later COVID-19 ICU hospitalizations. As reported by recent medical literature, the presence of these symptoms suggests that a follow-up program for these types of patients could be useful.

## 1. Introduction

The current global pandemic has had a dramatic impact on the healthcare system due to the high number of patients infected by coronavirus disease 2019 (COVID-19) admitted to hospitals and intensive care units (ICUs) [1,2]. Several medications and bundles of treatments have been sequentially proposed to reduce the high mortality rate and hospitalization [3,4]. COVID-19 triggers a harmful interleukin (IL) storm (IL-6, IL-1, and IL-18), so the use of agents against the cytokine storm, such as tocilizumab or anakinra, was introduced in Italy [3,4,5,6]. Tocilizumab, a monoclonal antibody against the interleukin 6 receptor, may counteract the syndrome of inflammatory cytokines released in patients with severe COVID-19 illness [7]. This paper aims to describe the one-year survival of ICU patients treated with tocilizumab compared with those who were not (treated versus untreated groups) in a single-center ICU cohort during the first COVID-19 wave.

## 2. Materials and Methods

We conducted an observational study approved by the local institutional ethics committee. From 29 February 2020 to 30 June 2020 (Protocol AOU 011422/20, Modena, Italy), the study enrolled all patients with confirmed COVID-19 diagnoses in our ICU (Baggiovara Hospital, Azienda Ospedaliero-Universitaria di Modena, Modena, Italy). We collected data using Microsoft Excel Software^®^ (Microsoft Corporation, 2018. Microsoft Excel). We assessed severity patients using ICU prognostic scores such as the sepsis-related organ failure assessment (SOFA) score and the simplified acute physiology score (SAPS II). We excluded all patients treated with different drugs against cytokine storm or cytokine removal systems and patients who failed to complete a one-year follow-up survival assessment. Each patient received a standard ICU treatment bundle according to the most recent guidelines for the care of patients infected by COVID-19. The bundle consisted of mechanical ventilation (all patients received mechanical ventilation, invasive or noninvasive); medical treatments with chloroquine, darunavir, vitamin C, N-acetylcysteine, and prophylaxis with low-molecular-weight heparin; and cortisone, administered only in confirmed or suspected pulmonary fibrosis cases. Remdesevir was unavailable in our center during the first COVID-19 wave: all Italian ICUs required many experimental drugs, which were not always available. We administered tocilizumab medication within the third day of ICU admission. The allocation of therapy was dependent on the availability of tocilizumab. The clinical contraindications to tocilizumab administration were the following conditions: known hypersensitivity to tocilizumab or its excipients, patients treated with immunomodulators or anti-rejection drugs, known active infections, bowel diverticulitis or perforation, and high level of serum transaminase, with a low level of platelets or neutrophils. Furthermore, we measured IL-6 at admission, at 48 h, and at 7 days after tocilizumab administration. On the first day, 8 mg/kg of tocilizumab was administered intravenously every 12 h (for a total of two doses) or once, with a subcutaneous injection of 162 mg. We also considered cortisone or therapeutic heparin treatment. We also recorded the multi-drug resistance (MDR) bacteria as possible complication due to tocilizumab treatment. We measured MDR as follows: methicillin-resistant Staphylococcus aureus (MRSA); vancomycin-resistant enterococci (VRE); extended-spectrum beta-lactamases (ESBL+); or bacteria with three or more antibiotic resistances in urinary, respiratory, or blood samples were classified as MDR infections. One year after ICU admission, we performed an accurate phone interview follow-up using the SF 36-item health survey questionnaire: we recorded any referred symptoms after one year of COVID-19 infection and ICU hospitalization, such as any possible complications, including paresthesia, neurological symptoms, pain, dyspnea, or other problems [8,9].

### Statistics

We analyzed the data with Stata^®^ software 16 (StataCorp, College Station, TX, USA). The results are expressed as mean (M) and standard deviation (SD), and the percentage of subjects was expressed as a percent of the total number of observations (obs), and the *p*-value was considered significant if <0.05. Confidence intervals (CIs) were calculated at 95%. All differences with a *p*-value (*p*) ≤ 0.05 (*) were considered statistically significant. We applied the Cox regression model to study the one-year follow-up by calculating the hazard ratio (Hr); continuous variables were tested with parametric and non-parametric tests. The research is an observational study: for that reason, we calculated treatment effects, with the propensity score matching two groups according to age and comorbidity scores using the Charlson Index: these last factors have a profound role in COVID-19 prognosis [10,11].

## 3. Results

**Figure 1:**
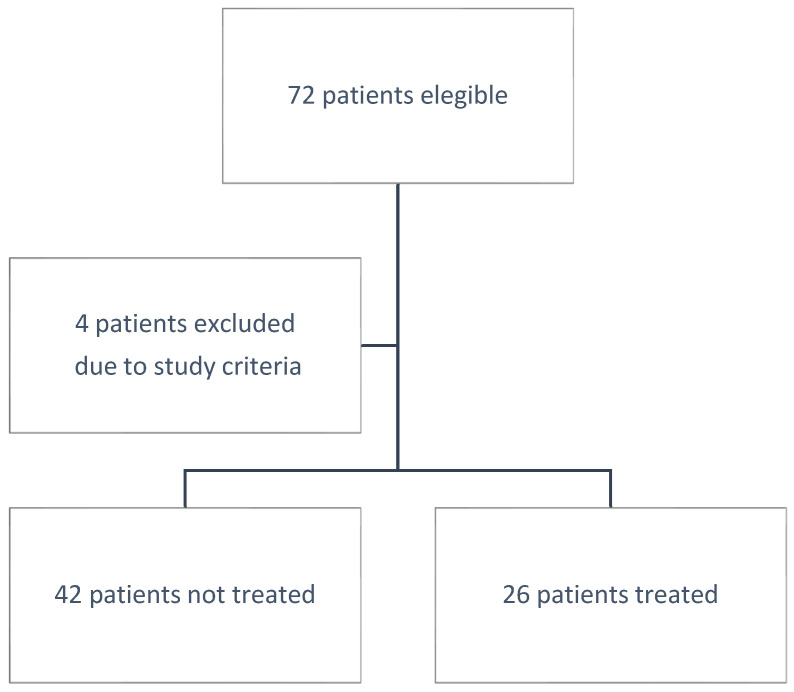
Study enrollment flowchart.

**Figure 2:**
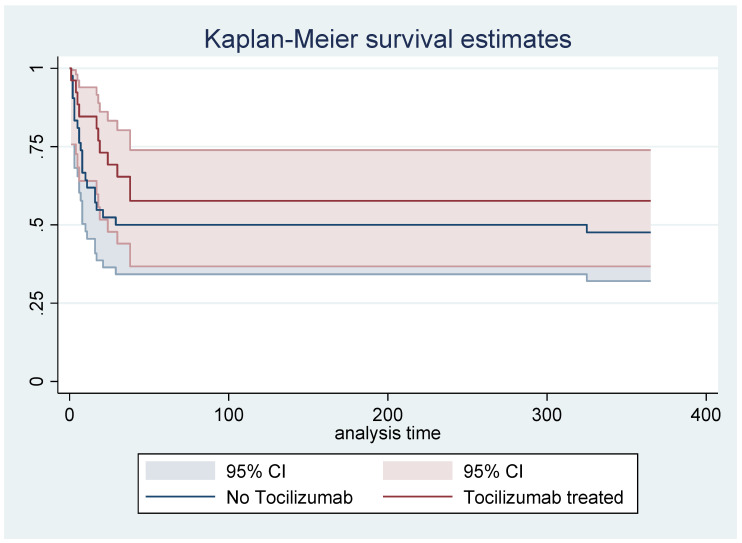
Hazard ratio of patients infected by COVID-19 in the ICU treated with tocilizumab: Hr 0.675 CI (0.32–1.39), *p*-value = 0.288.

We enrolled 68 ICU patients in our center on 72 occasions during the enrollment period; we excluded 4 patients: 3 patients treated with anakinra, and 1 patient with cytokine hemoadsorption (Figure 1). Of the patients, 38.24% (26 subjects) were in the tocilizumab group and the remaining were in the no-tocilizumab group (42 patients). The mean age was 64.38 ± 10.57; the tocilizumab group was younger than the no-tocilizumab group, with *p* = 0.009 *. The mean Charlson comorbidity index was lower in the treated group, with *p* 0.036. The SAPS II and SOFA scores at admission and 48 h later did not show any statistical differences. The cytokines levels at admission were not different; they were higher in the tocilizumab group at 48 h after admission and at one week after, with *p* 0.015 * and *p* 0.003 *, respectively. No significant differences were present between the groups in the cortisone treatment or therapeutic heparin treatments. The MDR rate was similar in both groups. The one-year mortality was not significantly lower in the tocilizumab group, and it did not reach any statistical significance. The one-year mortality hazard ratio of treated patients was 0.67 with a CI of 0.32 to 1.39, with *p* = 0.576 (Figure 2). The total number of patients who survived was 35 out of 68. Among the survivors, 32 of 35 patients answered the phone interview (14 patients in the treated group and 18 in the untreated group): 58.14% of patients reported a possible harmful effect of COVID-19 on their quality of life. The 39.39% did not blame any new-onset symptoms; the 33.33% referred to possible neurological symptoms such as paraesthesia, pain in the extremities, and difficulty in memory. The remaining 27.27% referred to symptoms of fatigue and dyspnea or to problems in the vocal cord related to tracheostomy or hospitalization. These symptoms were lower in the tocilizumab group (5/14 patients versus 14/18 patients) with *p* = 0.001 (Table 1 and Table 2).

## 4. Discussion

The first COVID-19 wave had a strong and terrible impact on the healthcare system in Italy, which was one of the first countries hit by the pandemic in Europe [12]. As in other disaster events, the Italian health care system provided a prompt response. Still, there was high mass media pressure, especially after the Bergamo town (Italy) experience, with an increasing number of critical and dead patients. There was significant pressure to find a strategy to reduce the mortality rate [13,14,15,16]. At the beginning of 2020, there was no substantial evidence for tocilizumab medication; nonetheless, the Food and Drug Agency and the European Medicines Agency authorized its administration based on the first positive experience and considering the events. With an increasing rate of ICU patients, the dramatic moment of the COVID-19 pandemic pushed many ICUs to start to use this agent against the cytokine storm to reduce the mortality rate in patients with the most severe COVID-19 infections [17,18,19,20]. This observational study describes and analyzes the use of tocilizumab in single-experience ICU patients during the first events of the pandemic after one year. The overall one-year mortality of patients infected by COVID-19 and admitted at the ICU in our center is slightly lower than the mortality described by Grasselli et al. (53.4%), who collected all data from ICUs of the Lombardia region, Italy, which was the region hit most severely by SARS-CoV-2 infections in the first pandemic wave [21]. Patients treated with tocilizumab showed lower, but not significant, mortality rates. This result is in accordance with the results presented by major trials and by the most significant trials; it seems that tocilizumab does not have a sufficiently positive effect on survival [5,22]. However, recent medical literature presents interesting positive experiences with tocilizumab, such as the study of Gupta et al., which describes how an early administration of tocilizumab could reduce the mortality rate, or the meta-analysis of Rezaei et al., where the results suggested that tocilizumab could improve clinical outcomes and survival [4,23]. We observed a lower (but not significant) mortality rate in our study, probably because the patients were younger and healthier than those not treated with tocilizumab and probably many additional reasons. First, tocilizumab was proposed as a possible medication against COVID-19 infection around the middle of March 2020; the outbreak in Italy started from the end of February, and the first patients admitted to the ICU who were infect by COVID-19 were older and had frail conditions. Furthermore, tocilizumab was not always available upon request: we did not administer the drug in cases with the presence of any health conditions that potentially increased the risk of infections or immunosuppression, selecting more healthy patients instead. However, no clinical differences were present in both groups upon admission: the SAPS II and SOFA scores were not statistically different, cortisone treatments or therapeutic heparin were applied equally in both groups, patients presented analog starting conditions, and patients showed the same percentage of MDR infections. Interestingly, our data highlighted how the cytokine storm does not reduce its plasma level after tocilizumab administration. Often, the cytokine levels remained higher in treated patients. A possible explanation for the higher IL-6 concentration may be that receptors were blocked by tocilizumab: if the receptors are blocked and bound, circulating IL-6 loses its effect. High levels of cytokines, especially IL-6, may have an adverse systemic impact, and tocilizumab could block this pathological pathway by binding IL-6 receptors [24,25]. Our data showed, a lower rate of referred new-onset symptoms at follow-up in the treated group. This result could arise from many conditions; as mentioned above, when treated patients were younger, the symptoms were only referred to and not clinically verified; in the end, the survivors’ sample size was small, so these results should be cautiously evaluated. Moreover, understanding and distinguishing the possible sequelae in these patients is fundamental, and a follow-up program could be useful, as recommended by Balachandar et al. [26]. It is important to remember that it is unknown how many potentially harmful effects could result from COVID-19 infection in people hit by this disease [27]. In our cohort, the rate of new-onset symptoms at follow-up are similar to the data shown by Taboada et al. and other authors [9,28,29]. In our cohort, the principal symptom that manifested was a neurological problem such as pain or paresthesia at the extremities, demonstrating that COVID-19 infection, starting through the angiotensin-converting enzyme 2 receptor (ACE2), develops into a systemic disease, with possible endothelium dysfunction, respiratory distress, and even neurological involvement [30,31,32,33,34,35]. The percentage of referred symptoms and clinical data reported are in accordance with the results of the largest follow-up [36,37,38]. In our cohort, the number of new-onset referred symptoms after COVID-19 infections are similar to the data of Taquet et al., but we also reported lower possible respiratory fatigue and abnormal breathing [39]. The group treated with tocilizumab showed a lower rate of overall new-onset symptoms. The meta-analysis by Wei et al. describes a possible positive effect of tocilizumab, especially for critical patients, and follow-up data of the largest and blinded clinical trials are still being conducted [40]. According to Stone et al., it is not possible to exclude that the treatments decreased the harmful inflammatory syndrome, but there is no evidence of a positive effect: further follow-up trials could clarify the value of this potential effect [41,42].

### Study Limitations

This study is a single-center observational study, but this study describes some important aspects of the first patients admitted to the ICU and infected by COVID-19 after one year. During the first COVID-19 wave, tocilizumab treatments were dedicated to the most critical patients, so it was difficult to make comparisons. We performed advanced statistical methods to improve the study design and are aware of our study’s limitations, but this study describes what really happened. Many ICUs upgraded and frequently changed their protocols, although the supply of tocilizumab medication was not always guaranteed due to the high number of patients. Patients referred their problems by phone interviews. The one-year follow-up coincided with the third COVID-19 wave, and it was not easy and safe to perform a face-to-face follow-up. In the non-administered group, the general condition may be more serious because there must be a reason as to why tocilizumab could not be administered. If so, it may be natural for symptoms to remain after treatment; for these reasons, we performed a propensity-score-matching treatment test. However, this type of study and the sample size of the survivors did not provide strong evidence. Thus, the results must be interpreted cautiously.

## 5. Conclusions

Our observational data followed the most relevant and largest trial. In our cohort, patients treated with tocilizumab referred to a lower rate of new-onset symptoms after COVID-19 ICU hospitalization; this result has to be weighted with the sample size of survivors, as different factors could relate to it. As reported by recent medical literature, the presence of these symptoms suggests that a follow-up program for these types of patients could be helpful, especially to better understand positive prognostic factors and treatments.

## Figures and Tables

**Table 1:** Principal clinical features, treatments, and complications between groups.

		Tocilizumab		
	Overall M ± SD or %	Treated M ± SD or %	Untreated M ± SD or %	*p*-Value
Age (years)	64.38 ± 10.57	60.19 ± 11.79	66.97 ± 8.93	0.009 *
Charlson Index (points)	3.54 ± 1.57	3.03 ± 1.56	3.87 ± 1.52	0.036 *
SOFA at admission (points)	3.67 ± 1.49	3.57 ± 1.57	3.73 ± 1.44	0.682
SOFA at 48 h from admission (points)	3.42 ± 2.15	3.24 ± 1.71	3.53 ± 2.41	0.593
SAPS II at admission (points)	32.94 ± 9.17	34.51 ± 9.90	30.46 ± 7.74	0.078
IL 6 in the first 24 h (pg/mL)	477.05 ± 626.91	462.27 ± 657.55	486.28 ± 615.34	0.882
IL 6 in the first 48 h (pg/mL)	519.16 ± 725.53	779.40 ± 900.80	287.60 ± 483.10	0.015 *
IL 6 at 7 days from admission (pg/mL)	632.51 ± 97.91	1034.37 ± 1212.40	212.40 ± 292.71	0.003 *
Cortisonic treatment (percentage (%))	52.94%	61.54%	47.62%	0.264
Therapeutic heparin (percentage (%))	41.18%	50%	35.71%	0.245
MDR infection intra-hospital (percentage (%))	41.18%	38.46	42.86	0.772
One-year mortality (percentage (%))	48.53%	42.31	52.38%	0.576 *
New-onset referred symptoms among survivors (percentage (%))	59.38%	35.71%	77.78%	0.001 *

**Table 2:** Onset of new reported symptoms after one year after COIVD-19 ICU hospitalization between groups.

		Follow-Up Survivors’ Treatment		
	Overall 33 (obs)	Treated (14 obs)	Untreated (19 obs)	
No new-onset symptoms reported	39.33%	57.14%	26.32%	
Referred neurological symptoms	33.33%	28.57%	36.84%	
Referred fatigue or dyspnea	9.09%	0.00%	15.79%	
Referred problems linked to COVID-19 hospitalization	18.80%	14.29%	21.05%	*p*-value 0.001 *

## Data Availability

Data are available upon request to the authors.

## References

[1] Huang C., Wang Y., Li X., Ren L., Zhao J., Hu Y., Zhang L., Fan G., Xu J., Gu X. (2020). Clinical features of patients infected with 2019 novel coronavirus in Wuhan, China. Lancet.

[2] Rivi V., Melegari G., Blom J.M.C. (2021). How to humanise the COVID-19 intensive care units. BMJ Evidence-Based Med..

[3] Zhang C., Wu Z., Li J.W., Zhao H., Wang G.Q. (2020). Cytokine release syndrome in severe COVID-19: Interleukin-6 receptor antagonist tocilizumab may be the key to reduce mortality. Int. J. Antimicrob. Agents.

[4] Gupta S., Wang W., Hayek S.S., Chan L., Mathews K.S., Melamed M.L., Brenner S.K., Leonberg-Yoo A., Schenck E.J., Radbel J. (2021). Association between Early Treatment with Tocilizumab and Mortality among Critically Ill Patients with COVID-19. JAMA Intern. Med..

[5] Salvarani C., Dolci G., Massari M., Merlo D.F., Cavuto S., Savoldi L., Bruzzi P., Boni F., Braglia L., Turrà C. (2021). Effect of Tocilizumab vs Standard Care on Clinical Worsening in Patients Hospitalized with COVID-19 Pneumonia: A Randomized Clinical Trial. JAMA Intern. Med..

[6] Samaee H., Mohsenzadegan M., Ala S., Maroufi S.S., Moradimajd P. (2020). Tocilizumab for treatment patients with COVID-19: Recommended medication for novel disease. Int. Immunopharmacol..

[7] Salama C., Han J., Yau L., Reiss W.G., Kramer B., Neidhart J.D., Criner G.J., Kaplan-Lewis E., Baden R., Pandit L. (2020). Tocilizumab in Patients Hospitalized with Covid-19 Pneumonia. N. Engl. J. Med..

[8] Apolone G., Mosconi P. (1998). The Italian SF-36 Health Survey: Translation, validation and norming. J. Clin. Epidemiol..

[9] Gamberini L., Mazzoli C.A., Sintonen H., Colombo D., Scaramuzzo G., Allegri D., Tonetti T., Zani G., Capozzi C., Giampalma E. (2021). Quality of life of COVID-19 critically ill survivors after ICU discharge: 90 days follow-up. Qual. Life Res..

[10] Gallo Marin B., Aghagoli G., Lavine K., Yang L., Siff E.J., Chiang S.S., Salazar-Mather T.P., Dumenco L., Savaria M.C., Aung S.N. (2021). Predictors of COVID-19 severity: A literature review. Rev. Med. Virol..

[11] Fang X., Li S., Yu H., Wang P., Zhang Y., Chen Z., Li Y., Cheng L., Li W., Jia H. (2020). Epidemiological, comorbidity factors with severity and prognosis of COVID-19: A systematic review and meta-analysis. Aging.

[12] Remuzzi A., Remuzzi G. (2020). COVID-19 and Italy: What next?. Heal. Policy.

[13] Barbieri A., Melegari G., Lob V., Mazzali L., D’Amelio L., Giovannoni A., Giuliani E. (2018). Response by Twin Italian Hub Hospitals in a Double Seismic Event: A Retrospective Observational Investigation. Prehospital Emerg. Care.

[14] Senni M. (2020). COVID-19 experience in Bergamo, Italy. Eur. Heart J..

[15] Fagiuoli S., Lorini F.L., Remuzzi G. (2020). Adaptations and Lessons in the Province of Bergamo. N. Engl. J. Med..

[16] Perico L., Tomasoni S., Peracchi T., Perna A., Pezzotta A., Remuzzi G., Benigni A. (2020). COVID-19 and lombardy: TESTing the impact of the first wave of the pandemic: The prevalence of SARS-CoV-2 infection in northern Italy. EBioMedicine.

[17] Melegari G., Giuliani E., Maini G., Barbieri L., Baffoni P., Bertellini E., Barbieri A. (2020). Novel coronavirus (2019-nCov): Do you have enough intensive care units?. Med. Intensiva.

[18] Grasselli G., Pesenti A., Cecconi M. (2020). Critical Care Utilization for the COVID-19 Outbreak in Lombardy, Italy: Early Experience and Forecast during an Emergency Response. JAMA—J. Am. Med. Assoc..

[19] Huang E., Jordan S.C. (2020). Tocilizumab for Covid-19—The Ongoing Search for Effective Therapies. N. Engl. J. Med..

[20] Toniati P., Piva S., Cattalini M., Garrafa E., Regola F., Castelli F., Franceschini F., Airò P., Bazzani C., Beindorf E.A. (2020). Tocilizumab for the treatment of severe COVID-19 pneumonia with hyperinflammatory syndrome and acute respiratory failure: A single center study of 100 patients in Brescia, Italy. Autoimmun. Rev..

[21] Grasselli G., Greco M., Zanella A., Albano G., Antonelli M., Bellani G., Bonanomi E., Cabrini L., Carlesso E., Castelli G. (2020). Risk Factors Associated With Mortality Among Patients With COVID-19 in Intensive Care Units in Lombardy, Italy. JAMA Intern. Med..

[22] Veiga V.C., Prats J.A.G.G., Farias D.L.C., Rosa R.G., Dourado L.K., Zampieri F.G., Machado F.R., Lopes R.D., Berwanger O., Azevedo L.C.P. (2021). Effect of tocilizumab on clinical outcomes at 15 days in patients with severe or critical coronavirus disease 2019: Randomised controlled trial. BMJ.

[23] Rezaei S., Fatemi B., Karimi Majd Z., Minaei H., Peikanpour M., Anjidani N., Taheri A., Dastan F., Mosaed R. (2021). Efficacy and safety of Tocilizumab in severe and critical COVID-19: A Systematic Review and Meta-Analysis. Expert Rev. Clin. Immunol..

[24] Nishimoto N., Terao K., Mima T., Nakahara H., Takagi N., Kakehi T. (2008). Mechanisms and pathologic significances in increase in serum interleukin-6 (IL-6) and soluble IL-6 receptor after administration of an anti–IL-6 receptor antibody, tocilizumab, in patients with rheumatoid arthritis and Castleman disease. Blood.

[25] Antwi-Amoabeng D., Kanji Z., Ford B., Beutler B.D., Riddle M.S., Siddiqui F. (2020). Clinical outcomes in COVID-19 patients treated with tocilizumab: An individual patient data systematic review. J. Med. Virol..

[26] Balachandar V., Mahalaxmi I., Subramaniam M., Kaavya J., Senthil Kumar N., Laldinmawii G., Narayanasamy A., Janardhana Kumar Reddy P., Sivaprakash P., Kanchana S. (2020). Follow-up studies in COVID-19 recovered patients—Is it mandatory?. Sci. Total Environ..

[27] Chippa V., Aleem A., Anjum F. (2021). Post Acute Coronavirus (COVID-19) Syndrome. StatPearls [Internet].

[28] Taboada M., Moreno E., Cariñena A., Rey T., Pita-Romero R., Leal S., Sanduende Y., Rodríguez A., Nieto C., Vilas E. (2021). Quality of life, functional status, and persistent symptoms after intensive care of COVID-19 patients. Br. J. Anaesth..

[29] Cortinovis M., Perico N., Remuzzi G. (2021). Long-term follow-up of recovered patients with COVID-19. Lancet.

[30] Zanza C., Tassi M.F., Romenskaya T., Piccolella F., Abenavoli L., Franceschi F., Piccioni A., Ojetti V., Saviano A., Canonico B. (2021). Lock, stock and barrel: Role of renin-angiotensin-aldosterone system in coronavirus disease 2019. Cells.

[31] None T.L.N. (2021). Long COVID: Understanding the neurological effects. Lancet Neurol..

[32] Melegari G., Rivi V., Zelent G., Nasillo V., De Santis E., Melegari A., Bevilacqua C., Zoli M., Meletti S., Barbieri A. (2021). Mild to severe neurological manifestations of covid-19: Cases reports. Int. J. Environ. Res. Public Health.

[33] Riva G., Nasillo V., Tagliafico E., Trenti T., Comoli P., Luppi M. (2020). COVID-19: More than a cytokine storm. Crit. Care.

[34] Zanza C., Racca F., Longhitano Y., Piccioni A., Franceschi F., Artico M., Abenavoli L., Maiese A., Passaro G., Volonnino G. (2021). Risk Management and Treatment of Coagulation Disorders Related to COVID-19 Infection. Int. J. Environ. Res. Public Health.

[35] Fisicaro F., Di Napoli M., Liberto A., Fanella M., Di Stasio F., Pennisi M., Bella R., Lanza G., Mansueto G. (2021). Neurological sequelae in patients with covid-19: A histopathological perspective. Int. J. Environ. Res. Public Health.

[36] Nature Medicine (2020). Meeting the challenge of long COVID. Nat. Med..

[37] The Lancet (2020). The Lancet Facing up to long COVID. Lancet.

[38] Del Rio C., Collins L.F., Malani P. (2020). Long-term Health Consequences of COVID-19. JAMA—J. Am. Med. Assoc..

[39] Taquet M., Dercon Q., Luciano S., Geddes J.R., Husain M., Harrison P.J. (2021). Incidence, co-occurrence, and evolution of long-COVID features: A 6-month retrospective cohort study of 273,618 survivors of COVID-19. PLoS Med..

[40] Wei Q., Lin H., Wei R.G., Chen N., He F., Zou D.H., Wei J.R. (2021). Tocilizumab treatment for COVID-19 patients: A systematic review and meta-analysis. Infect. Dis. Poverty.

[41] Tian J., Zhang M., Jin M., Zhang F., Chu Q., Wang X., Chen C., Yue H., Zhang L., Du R. (2021). Repurposed Tocilizumab in Patients with Severe COVID-19. J. Immunol..

[42] Stone J.H., Frigault M.J., Serling-Boyd N.J., Fernandes A.D., Harvey L., Foulkes A.S., Horick N.K., Healy B.C., Shah R., Bensaci A.M. (2020). Efficacy of Tocilizumab in Patients Hospitalized with Covid-19. N. Engl. J. Med..

